# *Akkermansia muciniphila* ameliorates fatty liver through microbiota-derived α-ketoisovaleric acid metabolism and hepatic PI3K/Akt signaling

**DOI:** 10.1016/j.isci.2025.112458

**Published:** 2025-04-16

**Authors:** Chang Liu, Rongrong Ma, Han Li, Xiaohua Pan, He Qian, Tianyi Yang, Yaoqi Tian

**Affiliations:** 1 State Key Laboratory of Food Science and Resources, Jiangnan University, 1800 Lihu Road, Wuxi 214122, China; 2 School of Food Science and Technology, Jiangnan University, 1800 Lihu Road, Wuxi 214122, China; 3 Analysis and Testing Center, Jiangnan University, Wuxi 214122, China

**Keywords:** Physiology, Molecular biology, Microbiology

## Abstract

*Akkermansia muciniphila* (Akk) has been shown to improve obesity via gut microbiota, while its effects on modulating gut fungi remain underexplored. This study investigates the effects of Akk on obese mice, focusing on gut fungi, metabolites, and hepatic lipid metabolism. We found that Akk treatment significantly modulated gut fungal diversity, enhanced gut immune responses, and improved fatty liver. Specifically, the abundance of harmful fungi *Fusarium* decreased. Subsequently, Akk improved hepatic lipid metabolism via the PI3K/Akt pathway, as determined by proteomics analysis. Additionally, an *in vitro* colonic organoid and microbiota co-culture system confirmed these effects by validating changes in key fungi and metabolites. Crucially, α-ketoisovaleric acid was identified as a pivotal metabolite, as its supplementation significantly improved hepatic lipid metabolism via PI3K/Akt pathway in obese mice. This study highlights Akk’s potential as a therapeutic agent for obesity by modulating gut fungi and identifying α-ketoisovaleric acid as a critical metabolite.

## Highlights

• Akk intervention decreased harmful fungi *Fusarium* and increased α-ketoisovaleric acid
• Akk improves lipid metabolism by the hepatic PI3K/Akt pathway
• Akk improves gut immune responses in organoids co-culture system
• α-ketoisovaleric acid improve the hepatic lipid metabolism in obese mice

## Graphical abstract

**Figure:**
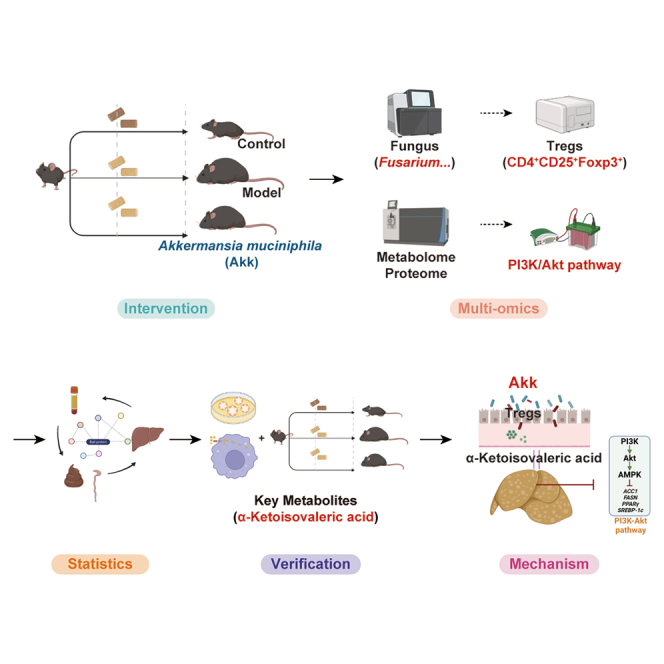

## Introduction

Obesity is a global health crisis characterized by excessive fat accumulation and associated with systemic inflammation and fatty liver.^1^ One of the key aspects of the pathogenesis of obesity is gut inflammation, which is significantly influenced by T cell-mediated immune responses.^2^ Regulatory T cells (Tregs) play a vital role in this context by modulating intestinal immune responses through multiple mechanisms: they secrete inhibitory cytokines, regulate metabolism, engage in cell-contact dependent inhibition, and help maintain the balance of gut microbiota.^3^ Understanding these immune mechanisms is crucial, especially as research increasingly reveals the complexity of the gut microbiome’s role in metabolic health.^4^ While much of the existing research has focused on the role of gut bacteria in modulating immune responses and overall metabolic health,^5^ the study of gut fungi has been relatively neglected. Gut fungi, though present in smaller numbers compared to bacteria, can have significant impacts on host metabolism and immune responses.^6^ They can interact with the host immune system and other microorganisms in the gut, influencing inflammatory processes and metabolic pathways.^7^ Therefore, a comprehensive understanding of gut microbiota’s role in obesity requires a closer look at the interactions between gut fungi, immune responses, and metabolic processes.

*Akkermansia muciniphila* (Akk), a mucin-degrading bacterium, has shown promise in improving metabolic health in obese and diabetic models.^8^ This bacterium achieves these beneficial effects by strengthening the mucus layer of the gut, which serves as a critical barrier against pathogenic bacteria and toxins.^9^ By regulating amino acid metabolism, Akk helps to prevent the translocation of harmful substances into the bloodstream, thereby reducing systemic inflammation and associated metabolic disorders.^10^ Despite these promising findings, there is limited research on how Akk influences gut fungi, the production of specific metabolites, and the modulation of inflammation. The gut microbiome is a complex ecosystem where bacteria and fungi coexist and interact with each other and with the host’s immune system.^11^ While much attention has been given to bacterial components, the fungal aspect remains relatively underexplored. Understanding how Akk affects gut fungi is essential. They can produce metabolites that influence the host’s immune responses and interact with other microbes, potentially affecting overall gut homeostasis.^12^

This study aims to evaluate the comprehensive effects of Akk on obese mice. We focus on changes in gut fungi and metabolomes, explore the modulation of gut immune responses, and examine the impact on hepatic lipid metabolism pathways in mice. To validate the role of gut fungi, we perform specific fungal depletion experiments to confirm their contribution to the observed effects. Furthermore, we investigate the role of specific metabolites regulated by Akk in reducing lipid accumulation in macrophages and compensation experiment in mice. Our study provides insights into the interactions between gut fungi, metabolites, and immune responses, offering a holistic understanding of Akk’s therapeutic potential in managing obesity.

## Results and discussion

### Akk improves fatty liver and systemic inflammation in obese mice

**Figure 1:**
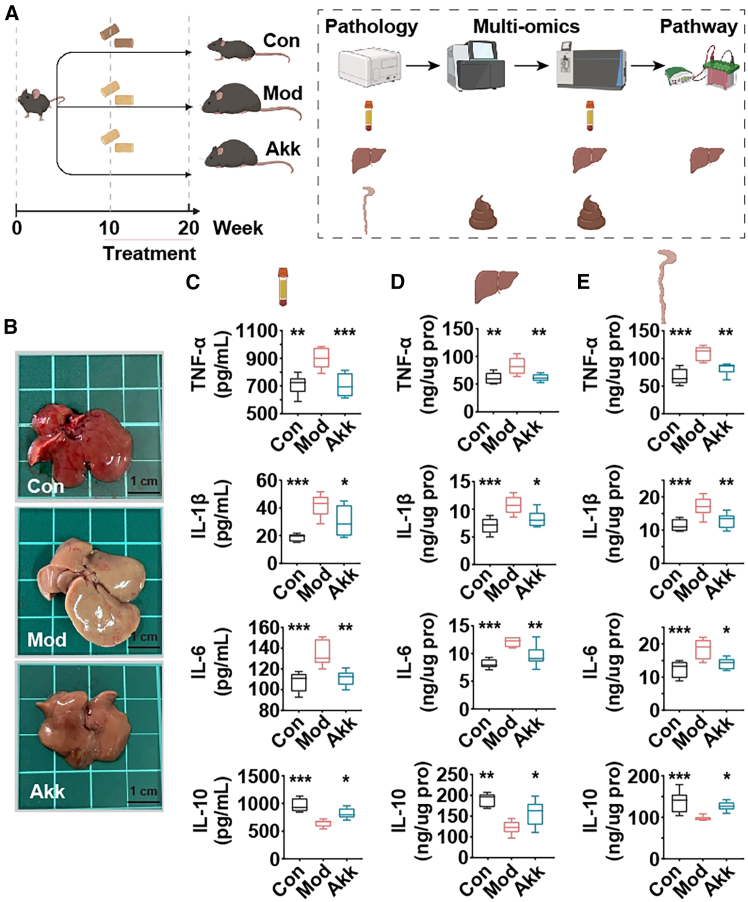
Akk Improves Fatty Liver and Systemic Inflammation in Obese Mice (A) Flowchart of the mice experiment detailing the experimental groups: control (Con), model (Mod), and Akk treatment (Akk). All mice, except for the Con group, were fed an HFHCD from week 1 to week 20. From week 11 to week 20, Akk group was orally administered bacterial suspension every day, while the Con and Mod groups were given saline. (B) Representative photographs of mouse livers from each group. (C) Serum levels of inflammatory cytokines TNF-α, IL-1β, IL-6, and anti-inflammatory cytokine IL-10. (D) Hepatic levels of inflammatory cytokines TNF-α, IL-1β, IL-6, and anti-inflammatory cytokine IL-10. (E) Colonic levels of inflammatory cytokines TNF-α, IL-1β, IL-6, and anti-inflammatory cytokine IL-10. Data expressed as mean ± SD (*n* = 8), ∗*p* < 0.05, ∗∗*p* < 0.01, ∗∗∗*p* < 0.001, compared with the Mod group, and *p* value was calculated using Dunnet’s post hoc test in one-way ANOVA. Scale bars: 1 cm.

The assessment of systemic inflammation and liver pathology is crucial in understanding the potential of nutritional intervention in obesity-related disorders.^13^ This study investigates the impact of Akk on inflammatory markers and liver condition in obese mice, providing insights into its mechanisms of action and potential benefits in managing obesity-induced inflammation and fatty liver. The experimental setup is illustrated in Figure 1A, where obese mice were divided into three groups: control (Con), model (Mod), and Akk (Akk) treatment. Photographs of the mouse livers (Figure 1B) demonstrate a marked improvement in liver appearance in the Akk-treated group compared to the Mod group, suggesting a reduction in fatty liver disease symptoms.

Obesity, particularly with an HFHCD, triggers systemic inflammation, which plays a pivotal role in exacerbating fatty liver disease. Inflammatory cytokines, such as TNF-α, IL-1β, and IL-6 are known to be upregulated in the liver and circulation in response to this chronic inflammation, leading to further liver fat accumulation.^14^ Inflammatory cytokine levels were measured in the serum (Figure 1C), liver (Figure 1D), and colon (Figure 1E) of the mice. In the serum, Akk significantly reduced the levels of TNF-α, IL-1β, and IL-6 while increasing IL-10 compared to the Mod group. Similar trends were observed in the hepatic and colonic tissues, with significant reductions in pro-inflammatory cytokines and an increase in the anti-inflammatory cytokine IL-10 in the Akk-treated group.

The reduction in TNF-α, IL-1β, and IL-6 levels, coupled with the increase in IL-10, suggests that Akk exerts anti-inflammatory effects, which may be beneficial in managing obesity-related inflammation and fatty liver disease. These findings align with previous studies highlighting the potential of Akk as a probiotic for improving metabolic health.^15^ The improvement in liver pathology and the anti-inflammatory effects observed in this study initially support the therapeutic potential of Akk in obesity and related metabolic disorders.

### Akk reshapes gut fungi in obese mice

Investigating the gut fungi is essential for understanding the complex interactions between host health and microbial communities.^6^ This study evaluates the impact of Akk on the gut fungal communities in obese mice, offering insights into its potential role in modulating gut microbiota and contributing to health benefits.

**Figure 2:**
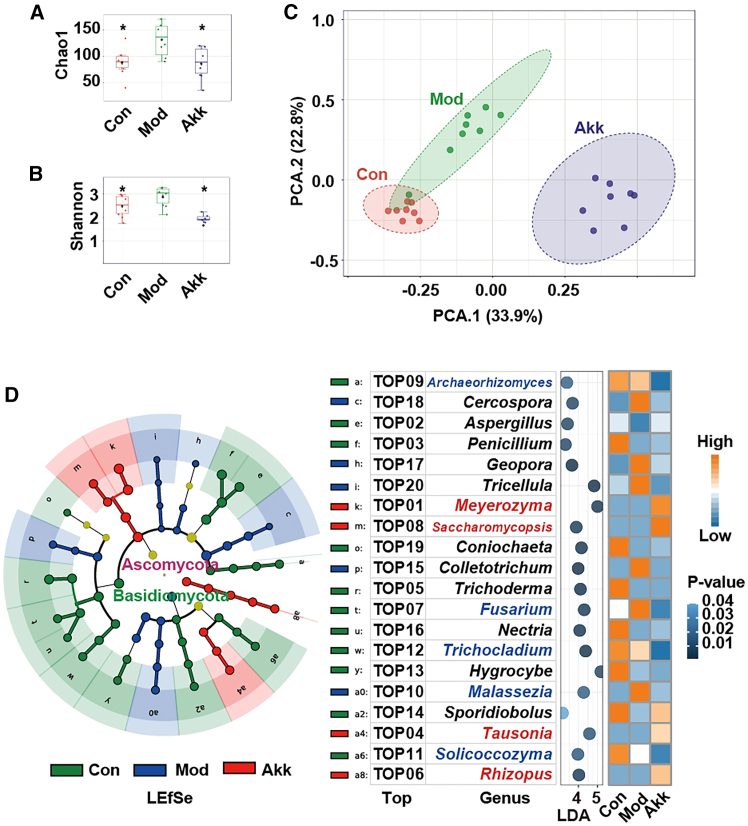
Akk reshapes gut fungi in obese mice (A) Chao1 index of fungi. (B) Shannon index of fungi. (C) PCA of fungal communities. (D) LEfSe analysis and heatmap of fungal taxa showing differential abundance among the groups. Data are expressed as mean ± SD (*n* = 8), ∗*p* < 0.05, ∗∗*p* < 0.01, compared with the Mod group, and *p* value was calculated using Dunnet’s post hoc test in one-way ANOVA. *p* < 0.01 and FDR <0.05 in LEfSe analysis.

The richness and diversity of the fungal communities were assessed using the Chao1 and Shannon indices, respectively. As shown in Figures 2A and 2B, Akk treatment significantly decreased both the richness and diversity of the gut fungal communities compared to the Mod group, indicating a more balanced fungal ecosystem, similar to the Con group. Principal component analysis (PCA) analysis (Figure 2C) revealed distinct clustering of the fungal communities among the Con, Mod, and Akk groups, suggesting that Akk profoundly alters the fungal composition in the gut. Compared with Mod group, Akk treatment led to a significant increase in *Saccharomycopsis* and a decrease in *Fusarium*. LefSe analysis (Figure 2D) further confirmed the differential abundance of specific fungal taxa among the groups. The heatmap highlights the taxa that were significantly enriched or depleted in response to Akk treatment. Notably, in the Akk group compared to the Mod group, there was an enrichment of fungi, such as *Meyerozyma*, *Saccharomycopsis*, *Tausonia*, and *Rhizopus*, whereas fungi, such as *Archaeorhizomyces*, *Fusarium*, *Trichocladium*, *Malassezia*, and *Solicoccozyma* were reduced. Specifically, *Saccharomycopsis* was enriched in the intestines of high-fat diet fed mice.^16^ Conversely, *Fusarium*, which primarily produces deoxynivalenol, is known to promote lipid metabolism disorders in obese mice induced by a high-fat diet.^17^ Furthermore, an increase in *Trichocladium* has been associated with patients suffering from primary sclerosing cholangitis with enteritis.^18^ *Malassezia* has been implicated in accelerating tumorigenesis in colon cancer patients.^19^ Lastly, a significant elevation of *Solicoccozyma* has been linked to the activation of pro-inflammatory IL-17 signaling.^20^ These findings underscore the diverse roles that specific fungal populations play in health and disease, highlighting their potential as targets.

Akk significantly reshapes the gut fungal communities in obese mice. The decrease in fungal richness and diversity, along with the beneficial shifts in fungal taxa, suggests that Akk may contribute to a healthier gut environment. The modulation of gut fungal communities by Akk may represent a mechanism through which this bacterium exerts its beneficial effects on host health.

### Akk improves gut immune responses in obese mice

**Figure 3:**
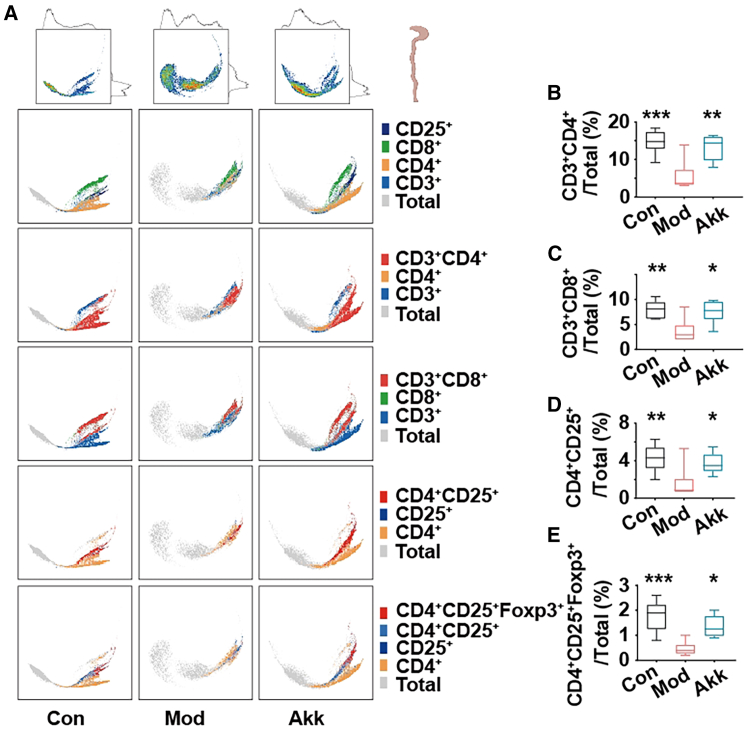
Akk improves gut immune responses in obese mice (A) PHATE of T cells clustering in mice intestinal lymph nodes. (B–E) Percentages of CD3^+^CD4^+^ Tregs, CD3^+^CD8^+^ Tregs, CD4^+^CD25^+^ Tregs, and CD4^+^CD25^+^Foxp3^+^ Tregs. Data are expressed as mean ± SD (*n* = 6), ∗*p* < 0.05, ∗∗*p* < 0.01, ∗∗∗*p* < 0.001, compared with the Mod group, and *p* value was calculated using Dunnet’s post hoc test in one-way ANOVA.

CD4^+^CD25^+^Foxp3^+^ Tregs regulate intestinal immune responses by secreting inhibitory cytokines, and maintaining gut microbiota balance.^21^^,^^22^ The impact of Akk on the intestinal T cell populations in obese mice was investigated to provide insights into its immunomodulatory effects in obesity. Figure 3 illustrates the effects of Akk on T cell populations in the intestine of obese mice. The PHATE dimensionality reduction plot (Figure 3A) shows distinct clustering of T cells in the Con, Mod, and Akk groups, indicating significant changes in T cell distribution with Akk treatment. The percentages of different Tregs subsets were quantified, revealing significant alterations. In the Akk-treated group, the percentage of CD3^+^CD4^+^ Tregs (Figure 3B) was significantly increased compared to the Mod group, indicating an enhancement in regulatory T cell populations which play a crucial role in maintaining immune homeostasis. Similarly, the percentage of CD3^+^CD8^+^ Tregs (Figure 3C) was significantly higher in the Akk group, suggesting a broader enhancement of regulatory T cell subsets. The CD4^+^CD25^+^ Tregs (Figure 3D) also showed a significant increase, further supporting the immunomodulatory effect of Akk. Foxp3^+^ Tregs are indispensable for maintaining gut immune system, where they play a pivotal role in modulating obesity-induced immune dysregulation. The pronounced effect was observed in the CD4^+^CD25^+^Foxp3^+^ Tregs (Figure 3E), where Akk treatment led to a substantial increase compared to the Mod group. Foxp3^+^ is a key marker of regulatory T cells, indicating that Akk significantly promotes the regulatory T cell lineage in the gut.

Previous research has highlighted the role of regulatory T cells in controlling intestinal inflammation and maintaining gut health.^23^ Akk significantly improves gut immune responses by enhancing the populations of regulatory T cells in obese mice. Notably, while Foxp3^+^ Tregs are essential in maintaining immune tolerance and modulating obesity-related inflammation, the broader enhancement of regulatory T cell subsets may also contribute to the observed effects. The increase in CD3^+^CD4^+^, CD3^+^CD8^+^, CD4^+^CD25^+^, and CD4^+^CD25^+^Foxp3^+^ Tregs suggests a comprehensive immunomodulatory effect of Akk, supporting its potential therapeutic application in managing obesity-induced inflammation and promoting gut health.

### Akk changes gut and serum metabolomes in obese mice

**Figure 4:**
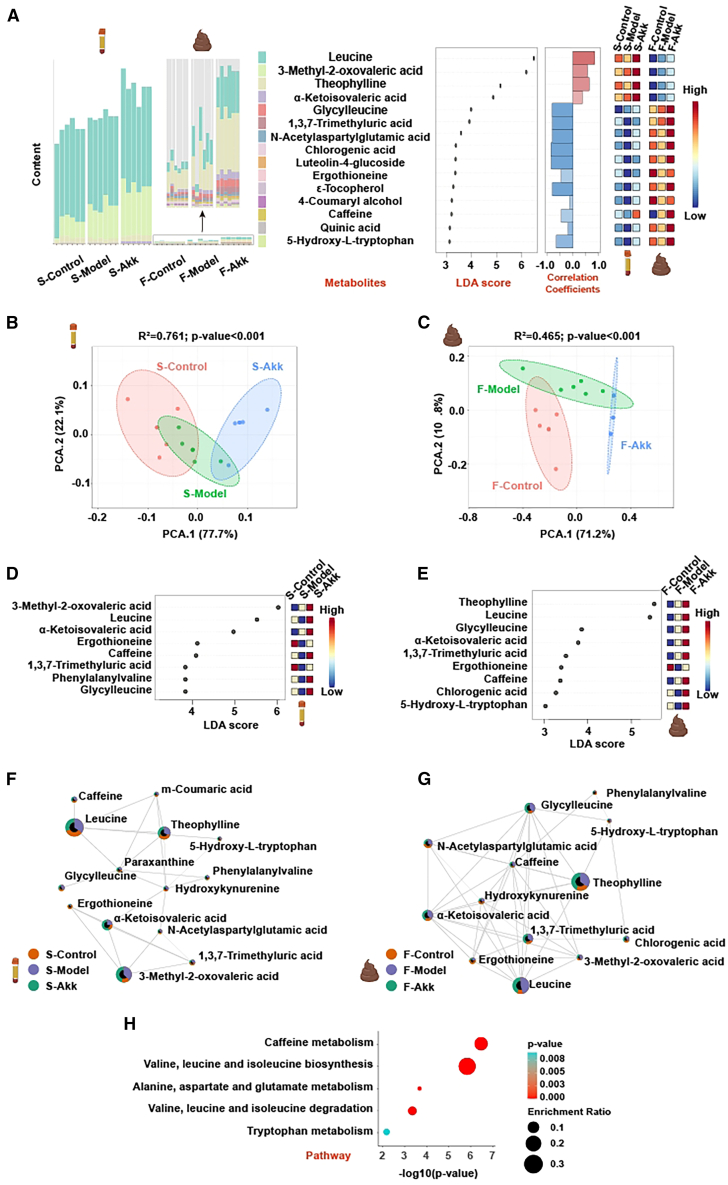
Akk changes gut and serum metabolomes in obese mice (A) Comprehensive analysis of metabolites in mouse serum and feces, including bar plots of content, LDA score plots, correlation coefficients, and heatmaps. (B and C) PCA of metabolites in mouse serum and feces. (D and E) LDA of metabolites in mouse serum and feces. (F and G) Network interaction analysis of metabolites in mouse serum and feces. (H) Pathway analysis of key metabolites in mouse serum and feces.

Comprehensive analysis of metabolites in mouse serum and feces (Figure 4A) revealed significant alterations in metabolic profiles between the control (S-control, F-control), model (S-model, F-model), and Akk-treated (S-Akk, F-Akk) groups. The content bar plots and linear discriminant analysis (LDA) score plots highlight the key metabolites that were differentially abundant across the groups. Correlation coefficients and heatmaps further illustrate the changes in metabolite levels, with notable increases in beneficial metabolites, such as leucine, 3-methyl-2-oxovaleric acid, theophylline, α-ketoisovaleric acid, glycylleucine, and chlorogenic acid in the Akk-treated group. PCA (Figures 4B and 4C) showed distinct separation between the groups, indicating that Akk significantly modulates the metabolic profiles in both serum and feces. The Akk-treated mice clustered separately from the Con group. LDA of serum and fecal metabolites (Figures 4D and 4E) and Network interaction analysis (Figures 4F and 4G) identified several key metabolites with significant differences in abundance, including leucine, α-ketoisovaleric acid, 3-methyl-2-oxovaleric acid, theophylline, and glycylleucine. 3-methyl-2-oxovaleric acid helps reduce obesity, increase energy expenditure, and improve glucose and insulin homeostasis.^24^ Theophylline is metabolized into caffeine in the liver and helps to reduce fat deposition and metabolic disorders induced by high-fat diet by regulating gut microbiota and caffeine metabolism.^25^

Valine, leucine, and isoleucine are known as branched-chain amino acids (BCAAs), and the catabolism of BCAAs after ingestion in the body generates branched-chain ketoacids, which are then gradually metabolized in the mitochondria to produce metabolites such as acetyl-CoA.^26^ It is worth noting that BCAAs-derived metabolite branched-chain α-ketoisovaleric acid (a metabolic intermediate of valine) regulates cell migration and proliferation, providing a target for clinical diagnosis and treatment targeting BCAA metabolic remodeling, and has important translational significance.^27^ The Akk-treated group showed enhanced connectivity among metabolites involved in crucial metabolic pathways, indicating a more integrated and balanced metabolic network. Pathway analysis (Figure 4H) highlighted the key metabolic pathways that were significantly impacted by Akk treatment. Pathways related to valine, leucine, and isoleucine biosynthesis and caffeine metabolism were significantly enriched. BCAAs also act as signaling molecules to regulate signaling pathways in the body, and their metabolic and perceptual imbalances play an important role in metabolic diseases, such as obesity.^28^ It suggests that Akk modulates these pathways to improve metabolic health in obese mice.

The ability of Akk to enhance amino acid metabolism suggests potential mechanisms through which it improves metabolic homeostasis and reduces obesity-related metabolic disturbances.

### Akk improves hepatic lipid metabolism via PI3K/Akt pathway in obese mice

**Figure 5:**
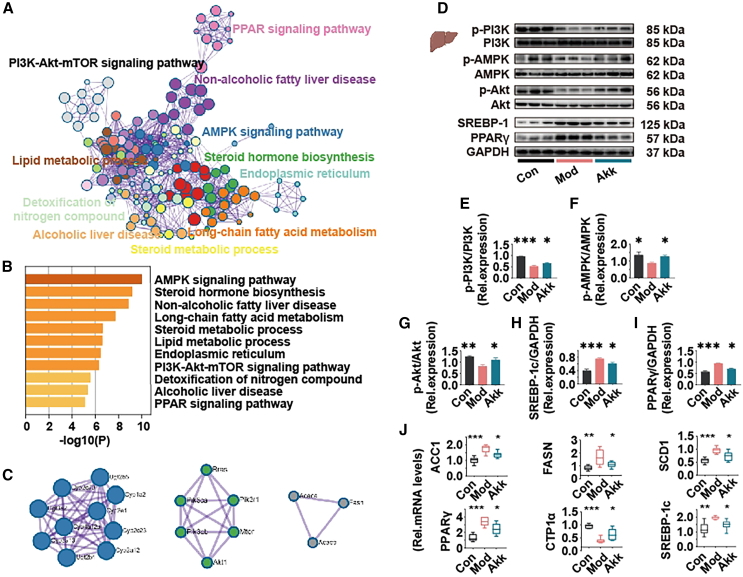
Akk improves hepatic lipid metabolism via PI3K/Akt pathway in obese mice (A–C) Akk significantly affected mouse liver protein network interaction pathways, significantly regulated pathways and differential proteins by proteomic analysis. (D–J) Western blot in the PI3K/Akt pathway in mouse liver: p-PI3K/PI3K, *p*-AMPK/AMPK, *p*-Akt/Akt, PPARγ/GAPDH, and SREBP-1c/GAPDH. (K) mRNA levels of genes related to lipogenesis (ACC1, FASN, SCD1, and PPARγ), lipid oxidation (CPT1α), and lipid uptake (SREBP-1c) in mouse liver. Data are expressed as mean ± SD (n = 6–8), ∗*p* < 0.05, ∗∗*p* < 0.01, ∗∗∗*p* < 0.001, compared with the Mod group, and *p* value was calculated using Dunnet’s post hoc test in one-way ANOVA.

To investigate the impact of Akk treatments on liver metabolism, we conducted a proteomic analysis focusing on differentially expressed proteins within relevant pathways. Search tool for the retrieval of interacting genes/proteins (STRING) enrichment website (https://cn.string-db.org/) was used for protein-protein interaction enrichment analysis. (Figure S1) Subsequently, using complementary tools (DAVID and Metascape) and databases (GO, Kyoto encyclopedia of genes and genomes (KEGG), and Reactome), we performed functional annotation, identifying significant pathway modulations (*p* < 0.05). As shown in Figure 5A, key metabolic pathways, including PI3K/Akt signaling, AMPK signaling, and lipid metabolism, were prominently enriched. Notably, proteins associated with the AMPK signaling pathway were among the most significantly regulated, indicating enhanced metabolic activity (Figure 5B). Protein-protein interaction analysis (Figure 5C) revealed intricate networks, highlighting the interconnected nature of lipid metabolism and PI3K/Akt pathway. The findings suggest that these pathways play a critical role in liver function modulation, with potential implications for Akk interventions targeting metabolic disorders.

PI3K/Akt signaling pathway plays a critical role in regulating lipid metabolism, and its dysregulation is associated with obesity and fatty liver disease.^29^^,^^30^ Analysis of key proteins involved in the PI3K/Akt pathway (Figures 5D–5J) revealed significant changes in their expression levels following Akk treatment. The ratios of phosphorylated PI3K (p-PI3K/PI3K) (Figure 5E), AMPK (*p*-AMPK/AMPK) (Figure 5F), and Akt (*p*-Akt/Akt) (Figure 5G) were significantly increased in the Akk-treated group, indicating activation of p-PI3K, *p*-AMPK, and *p*-Akt. The expression of SREBP-1c (Figure 5H) and PPARγ (Figure 5I), key regulators of lipid metabolism, were significantly reduced in the Akk-treated group, indicating a potential reduction in lipogenesis and lipid uptake.^31^^,^^32^ SREBP is a transcription factor that controls cholesterol and fatty acid metabolism.^32^ The mRNA levels of genes related to lipogenesis (ACC1, FASN, SCD1, and PPARγ) and lipid uptake (SREBP-1c) were significantly reduced in the Akk-treated group, while the expression of CPT1α, a gene involved in lipid oxidation, was significantly increased (Figure 5J). In the liver (Figure S3A) and colon (Figure S3B), the mRNA levels of the CD36 gene show that intervention with Akk significantly reduced CD36 expression in the liver, while there was no significant effect on CD36 expression in the colon. This suggests that Akk primarily influences hepatic lipid metabolism, with limited impact on intestinal lipid absorption. These changes suggest a shift toward enhanced lipid oxidation and reduced lipogenesis and lipid uptake in the liver. Although Akt and AMPK are generally regarded as having opposing effects on metabolic homeostasis, recent studies have demonstrated that they can be co-activated under certain conditions. For example, IL-22 has been shown to induce the activation of both Akt and AMPK, thereby regulating hepatocyte metabolism and promoting lipid oxidation.^33^

The regulation of the PI3K/Akt pathway indicate a shift toward reduced lipogenesis, contributing to improved liver health in obese mice.^34^ Akk improves hepatic lipid metabolism by modulating the PI3K/Akt signaling pathway.

### Akk mediates gut immunity through metabolites to improve hepatic lipid metabolism

**Figure 6:**
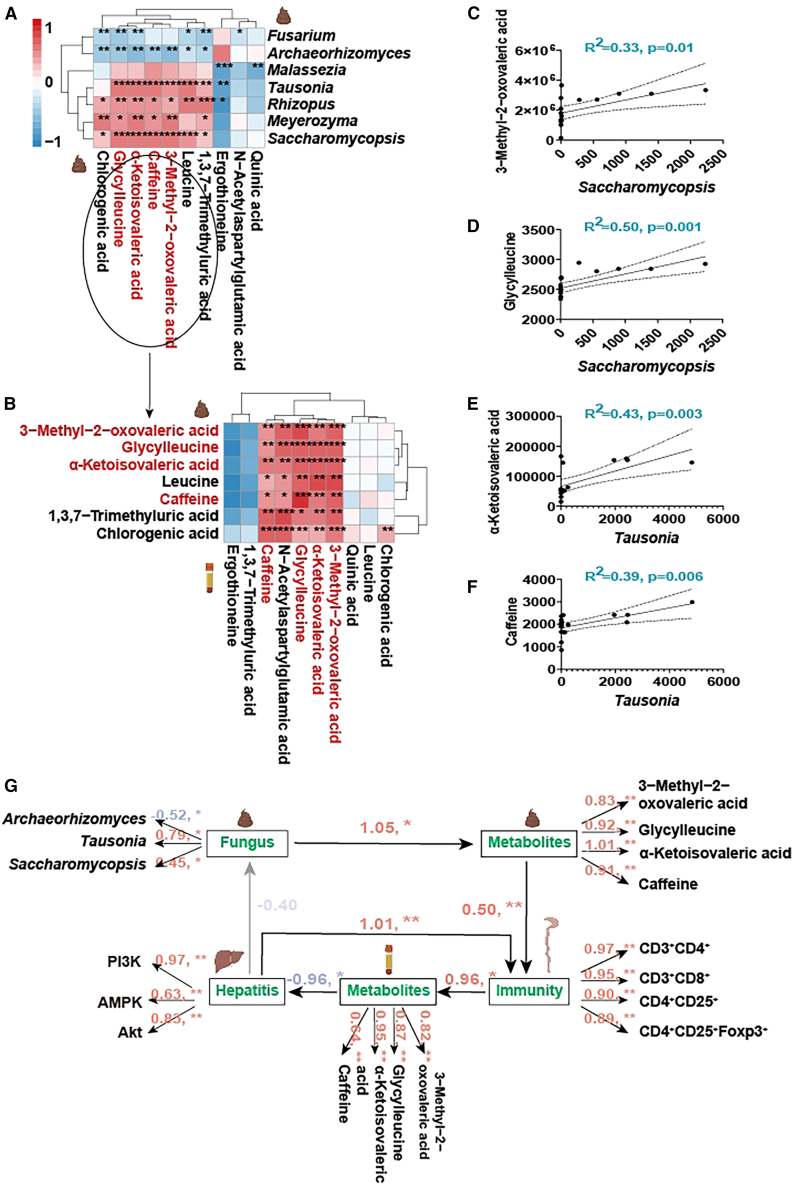
Akk mediates gut immunity through metabolites to improve hepatic lipid metabolism (A and B) Correlation analysis between gut fungi and gut metabolites, and between gut metabolites and serum metabolites. (C and D) Regression analysis of 3-methyl-2-oxovaleric acid and glycylleucine with *Saccharomycopsis*. (E and F) Regression analysis of α-ketoisovaleric acid and caffeine with *Tausonia*. (G) Pathway analysis under structural equation modeling illustrating the “gut fungi-gut metabolites-gut immunity-serum metabolites-fatty liver” pathway. ∗*p* < 0.05, ∗∗*p* < 0.01, ∗∗∗*p* < 0.001 (*n* = 8).

The intricate connections between gut microbiota, metabolites, immunity, and hepatic health is crucial for developing comprehensive strategies to combat obesity-related liver diseases.^35^ The mediatory role of metabolites in the relationship between gut fungi and immunity was investigated, and their ultimate impact on hepatic lipid accumulation, providing a holistic view of Akk’s beneficial effects. Correlation analyses between gut fungi and gut metabolites (Figure 6A) and between gut and serum metabolites (Figure 6B) revealed significant associations. Initially, a significant correlation was observed between gut fungi and several metabolites, including chlorogenic acid, glycylleucine, α-ketoisovaleric acid, caffeine, 3-methyl-2-oxovaleric acid, leucine, and 1,3,7-trimethyluric acid (Figure 6A). Next, based on the correlation between gut and serum metabolites, chlorogenic acid, leucine, and 1,3,7-trimethyluric acid were excluded due to the lack of significant correlations (Figure 6B). Consequently, the remaining four metabolites were further analyzed.

Regression analysis further highlighted these associations, with 3-methyl-2-oxovaleric acid (R^2^ = 0.33, *p* = 0.01) and glycylleucine (R^2^ = 0.5, *p* = 0.001) showing strong positive correlations with *Saccharomycopsis* (Figures 6C and 6D), and α-ketoisovaleric acid (R^2^ = 0.43, *p* = 0.003) and caffeine (R^2^ = 0.39, *p* = 0.006) correlating significantly with *Tausonia* (Figures 6E and 6F). Figure S2 presents the regression analyses between key metabolites and various gut fungi. The analyses demonstrate significant negative correlations between *Archaeorhizomyces* and metabolites, such as 3-methyl-2-oxovaleric acid. Similar patterns were observed with *Saccharomycopsis*, where α-ketoisovaleric acid exhibited significant positive correlations.

Structural equation modeling (Figure 6G) elucidated the comprehensive pathway from gut fungi to improved hepatic lipid accumulation via metabolites and immune modulation. The model demonstrated Akk modulates gut fungi, which in turn influence gut metabolites. These metabolites affect gut immunity, ultimately leading to changes in serum metabolites and resulting in reduced hepatic lipid accumulation. By modulating key metabolites and enhancing gut immunity, Akk contributes to a reduction in hepatic lipid accumulation and improved liver health in obese mice.

### Gut fungi is the key in Akk improving gut immune responses in organoids

**Figure 7:**
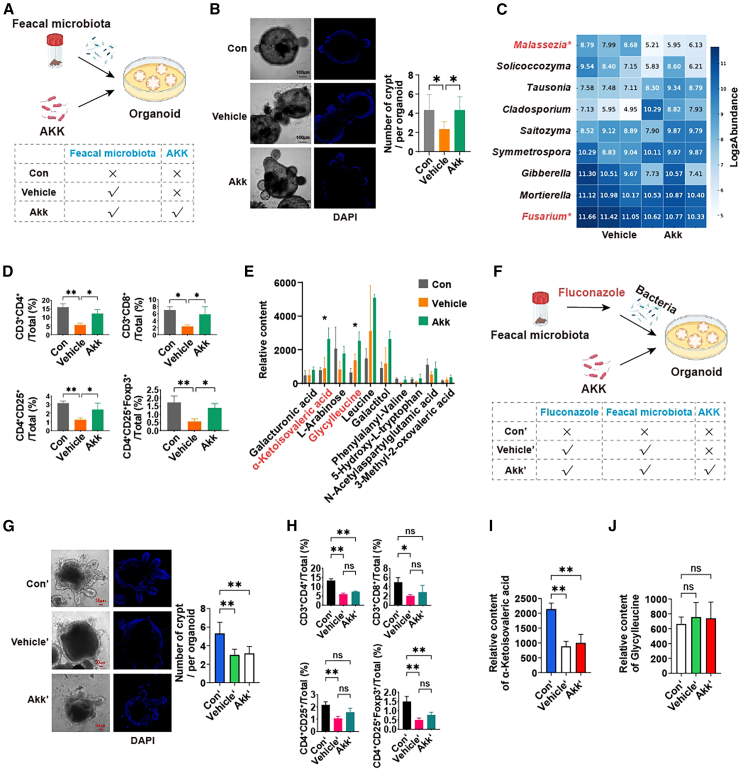
Gut fungi is the key in Akk improving gut immune responses in organoids (A) Co-culture of colonic organoids, fecal microbiota and Akk. (B) Fluorescent images of colonic organoids with DAPI-labeled cells, and number of organoid crypts. Scale bars: 100 μm. (C) Relative abundance of fungal in co-culture system. (D) Percentages of CD3^+^CD4^+^, CD3^+^CD8^+^, CD4^+^CD25^+^, and CD4^+^CD25^+^Foxp3^+^ Tregs in organoids. (E) Relative content of metabolites in co-culture system. (F) Co-culture of colonic organoids, fluconazole, fecal microbiota and Akk. (G) Fluorescent images of colonic organoids with DAPI-labeled cells, and number of organoid crypts. Scale bars: 50 μm. (H) Percentages of CD3^+^CD4^+^, CD3^+^CD8^+^, CD4^+^CD25^+^, and CD4^+^CD25^+^Foxp3^+^ Tregs in organoids. (I and J) Relative content of metabolites (α-ketoisovaleric acid and glycylleucine) in co-culture system. Figures 7A–7E and Figures 7F–7J represent experiments from different batches, respectively. Data are expressed as mean ± SD (n = 6–8), ^ns^p >0.05, ∗*p* < 0.05, ∗∗*p* < 0.01, ∗∗∗*p* < 0.001, compared with the Mod group, and *p* value was calculated using Dunnet’s post hoc test in one-way ANOVA.

To validate the effects of Akk on human gut fungi and intestinal immunity *in vitro*, we conducted the co-culture system (Figure 7A), involving colonic organoids, fecal microbiota, and Akk. We observed that co-culture with Akk led to a notable enhancement in the generation of colonic organoid crypts (Figure 7B), which is indicative of improved intestinal epithelial renewal. This finding is consistent with *in vivo* observations in mice, where a reduction in the pathogenic fungus *Fusarium* was identified (Figure 7C). Furthermore, an upregulation of CD4^+^CD25^+^Foxp3^+^ Tregs highlights a significant improvement in intestinal immune function (Figure 7D). These results highlight that Akk not only reduces harmful fungi but also enhances gut immunity.

To further validate the metabolic effects of Akk on the human gut microbiota, we conducted a comprehensive metabolite analysis. Notably, two metabolites, glycylleucine and α-ketoisovaleric acid, were significantly elevated in our co-culture system (Figure 7E). These findings mirror the results observed in mice, suggesting that Akk exerts a similar metabolic influence on the gut microbiota in humans. This metabolite profiling underscores the functional importance of Akk in shaping gut microbial metabolism and supports its potential therapeutic role in modulating gut health.

Next, we investigated the critical role of gut fungi in mediating the effects of Akk on gut immunity and metabolite production using a co-culture model with colonic organoids, fecal microbiota, and fluconazole-mediated fungal depletion (Figure 7F). Our results demonstrate that, in the absence of gut fungi, Akk was unable to promote crypt formation in the organoids, as shown in Figure 7G. Organoids cultured with fluconazole-treated microbiota did not exhibit an increase in the number of crypts, unlike the control conditions where gut fungi were present. Moreover, fluconazole treatment prevented the expected improvements in gut immune responses. In Figure 7H, flow cytometry analysis revealed no significant changes in the percentages of CD3^+^CD4^+^, CD3^+^CD8^+^, CD4^+^CD25^+^, and CD4^+^CD25^+^Foxp3^+^ Tregs in organoids co-cultured with fluconazole-treated microbiota and Akk. This absence of immune modulation highlights the essential role of gut fungi in Akk’s beneficial effects on gut immunity.

Finally, we investigated the production of key metabolites, such as α-ketoisovaleric acid and glycylleucine, known to be important in metabolic regulation. As shown in Figures 7I and 7J, the relative content of α-ketoisovaleric acid was significantly reduced in organoids co-cultured with fluconazole-treated microbiota, and no significant increase in glycylleucine was observed. These findings suggest that the production of the crucial metabolite, α-ketoisovaleric acid, is heavily dependent on the presence of gut fungi. Specifically, in the absence of gut fungi, Akk was unable to promote immune responses, induce crypt formation in colonic organoids, or generate significant levels of α-ketoisovaleric acid. This highlights the essential role of gut fungi in enabling Akk to facilitate these processes and produce key metabolites.

### Key metabolites regulated by Akk improve lipid metabolism in macrophages

Macrophages will be transformed into foam cells after ingesting excessive ox-LDL, resulting in the disturbance of lipid metabolism.^36^ Lipid homeostasis in macrophages mainly includes lipid uptake, esterification, and outflow.^37^ To elucidate the potential intervention mechanisms of key metabolites affected by Akk in obesity-related inflammation, the effects of glycylleucine and α-ketoisovaleric acid on macrophage lipid accumulation and inflammatory response were finally evaluated.

**Figure 8:**
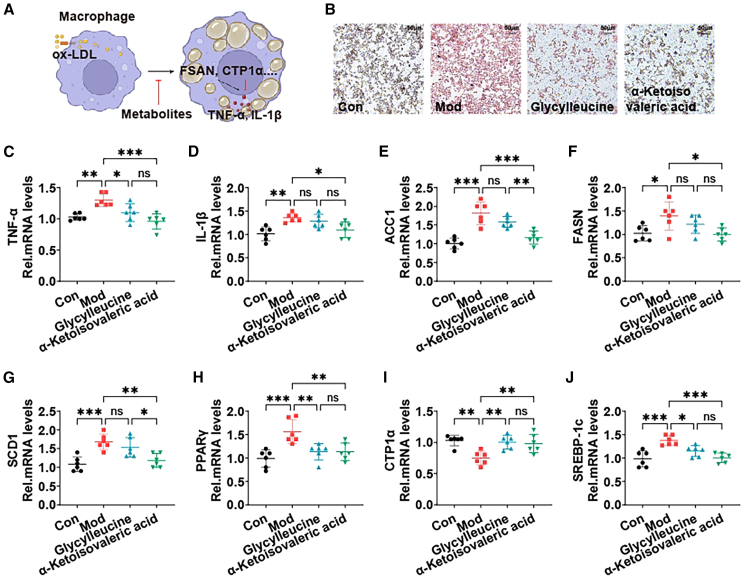
Key metabolites regulated by Akk improve lipid metabolism in macrophages (A) Schematic of the macrophage lipid accumulation model and co-culture with metabolites. (B) Oil red O staining of cells to visualize lipid accumulation. (C and D) mRNA levels of inflammatory cytokines TNF-α and IL-1β in cells. (E–J) mRNA levels of genes related to lipogenesis (ACC1, FASN, SCD1, and PPARγ), lipid oxidation (CPT1α), and lipid uptake (SREBP-1c) in cells. Data are expressed as mean ± SD (*n* = 6), ^ns^p >0.05, ∗*p* < 0.05, ∗∗*p* < 0.01, ∗∗∗*p* < 0.001, compared with the Mod group, and *p* value was calculated using Dunnet’s post hoc test in one-way ANOVA. Scale bars: 50 μm.

Figure 8A depicts the experiment where macrophages were co-cultured with key metabolites to assess their effects on lipid accumulation. Oil red O staining of the cells shows a significant reduction in lipid accumulation in the glycylleucine and α-ketoisovaleric acid treated groups compared to the Mod group, indicating that these metabolites can mitigate lipid accumulation in macrophages (Figure 8B). The mRNA levels of inflammatory cytokines TNF-α and IL-1β were significantly reduced in the metabolite-treated groups, suggesting that glycylleucine and α-ketoisovaleric acid can also alleviate inflammation in macrophages (Figures 8C and 8D). In addition, α-ketoisovaleric acid reduces the expression of inflammatory factors to promote lipid outflow, which helps to reduce cellular lipid accumulation.

Excessive ox-LDL destroys cell homeostasis, and the metabolites of intestinal microbiota in mice after Akk intervention maintain lipid homeostasis of macrophages through PPARγ.^38^ The expression of genes related to lipogenesis (ACC1, FASN, SCD1, and PPARγ) and lipid uptake (SREBP-1c) was significantly downregulated in the metabolite-treated groups. Conversely, the expression of CPT1α, a gene involved in lipid oxidation, was significantly upregulated, indicating an enhanced lipid oxidation process (Figures 8E–8J).^39^ α-ketoisovaleric acid was more significant than glycylleucine, while glycylleucine was not significant in TNF-α, ACC1, FASN, and SCD1.

Therefore, key metabolites, especially α-ketoisovaleric acid, regulated by Akk significantly reduce lipid accumulation and inflammatory responses in macrophages, further supporting the mechanisms through which Akk-derived metabolites exert their beneficial effects on obesity.

### α-ketoisovaleric acid is the key metabolite in improving hepatic lipid metabolism via PI3K/Akt pathway in obese mice

**Figure 9:**
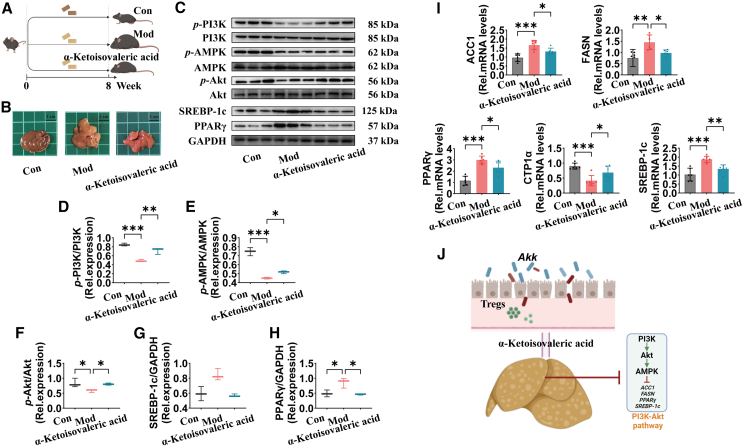
α-ketoisovaleric acid is the key metabolite in improving hepatic lipid metabolism via PI3K/Akt pathway in obese mice (A) Flowchart of α-ketoisovaleric acid compensation experiment. All mice, except for the Con group, were fed an HFHCD from week 1 to week 8. α-ketoisovaleric acid group was orally administered α-ketoisovaleric acid every day, while the Con and Mod groups were given saline. (B) Representative photographs of mouse livers from each group. (C–H) Western blot in the PI3K/Akt pathway in mouse liver: p-PI3K/PI3K, *p*-AMPK/AMPK, *p*-Akt/Akt, PPARγ/GAPDH, and SREBP-1c/GAPDH. (I) mRNA levels of genes related to lipogenesis (ACC1, FASN, and PPARγ), lipid oxidation (CPT1α), and lipid uptake (SREBP-1c) in mouse liver. (J) Akk improves hepatic lipid metabolism via PI3K/Akt pathway through α-ketoisovaleric acid. Data are expressed as mean ± SD (n = 6–8), ∗*p* < 0.05, ∗∗*p* < 0.01, compared with the Mod group, and *p* value was calculated using Dunnet’s post hoc test in one-way ANOVA. Scale bars: 1 cm.

Previous studies have identified α-ketoisovaleric acid as a key metabolite regulated by Akk. Our experimental design (Figure 9A) involved compensating mice with α-ketoisovaleric acid over an 8-week period, assessing its impact on liver morphology and molecular pathways involved in lipid metabolism. Photographic evidence (Figure 9B) showed that α-ketoisovaleric acid treatment resulted in visibly healthier liver tissue compared to the Mod group, indicating a reduction in hepatic steatosis. Western blot analysis (Figures 9C–9H) revealed the effects of α-ketoisovaleric acid on the PI3K/Akt pathway in mouse liver tissues. α-ketoisovaleric acid treatment significantly changed the phosphorylation of key proteins. p-PI3K/PI3K ratio was elevated, suggesting increased PI3K pathway activity. *p*-AMPK/AMPK ratio and *p*-Akt/Akt ratio showed significant upregulation, highlighting improved energy homeostasis. PPARγ/GAPDH ratio and SREBP-1c/GAPDH ratio were both significantly decreased, which are indicative of improved lipid metabolism.

Quantitative PCR analysis further supported these findings by showing increased mRNA levels of genes associated with lipogenesis (ACC1, and FASN), lipid oxidation (CPT1α), and lipid uptake (SREBP-1c) in the α-ketoisovaleric acid-treated group compared to the Mod group (Figure 9I). These gene expression changes correlate with enhanced lipid metabolic processes and reduced hepatic lipid accumulation. Figure 9J illustrates the proposed mechanism by which Akk exerts its beneficial effects on hepatic lipid metabolism through the modulation of the PI3K/Akt pathway via α-ketoisovaleric acid. Following intervention with α-ketoisovaleric acid, a significant reduction in CD36 gene expression was observed in the liver, whereas no significant changes were detected in the colon (Figures S3C and S3D). This further demonstrates that α-ketoisovaleric acid mainly regulates hepatic lipid metabolism. This metabolite appears to play a critical role in mediating the beneficial effects of Akk, promoting Tregs and enhancing hepatic lipid metabolism. Thus, the compensation experiments further demonstrated that α-ketoisovaleric acid is a key metabolite produced in the presence of gut fungi, and it plays a crucial role in Akk-mediated improvement of hepatic lipid metabolism.

Thus, this study comprehensively investigates the beneficial effects of Akk on obese mice, focusing on gut fungi, gut immune responses, and hepatic lipid metabolism. Our findings demonstrate that Akk treatment significantly decreases harmful fungi like *Fusarium*, enhances gut immune responses, and improves fatty liver through the PI3K/Akt pathway. Proteomics analysis further identified key signaling pathways impacted by Akk, corroborating the observed changes in fungal populations and immune responses. Additionally, key metabolites regulated by Akk, such as glycylleucine and α-ketoisovaleric acid, were shown to reduce lipid metabolism and inflammatory responses in macrophages. The supplementation of α-ketoisovaleric acid notably improved hepatic lipid metabolism via the PI3K/Akt pathway, highlighting its role as a pivotal metabolite in the therapeutic effects observed. The results from *in vitro* colonic organoid and microbiota co-culture further validated these findings, revealing consistent effects on key fungi and metabolites. Overall, our study underscores the intricate interactions between gut fungi, metabolites, and the therapeutic benefits induced by Akk.

### Limitations of the study

While the effects of Akk on obese mice are promising, the findings may not fully translate to human physiology, and further clinical studies are needed to confirm whether these therapeutic benefits extend to humans via this pathway. Additionally, the study lacks validation through liver-targeted gene knockouts, which would help confirm the specific role of identified signaling pathways and metabolites in hepatic lipid metabolism and immune modulation. Moreover, the use of fecal samples from only male human donors and the exclusive use of male mice limit the generalizability of the findings across sexes.

## Resource availability

### Lead contact

Requests for further information and resources should be directed to and will be fulfilled by the lead contact, Yaoqi Tian (yqtian@jiangnan.edu.cn).

### Materials availability

This study did not generate any new or unique reagents.

### Data and code availability

• The data supporting the current study have been deposited at Mendeley Data and are publicly available as of the date of publication. Accession numbers are listed in the key resources table.
• This paper does not report original code.
• Any additional information required to reanalyze the data reported in this paper is available from the lead contact upon request.

## Acknowledgments

This work is financially supported by 10.13039/501100002858China Postdoctoral Science Foundation (2024M761182), Postdoctoral Fellowship Program of CPSF (no.GZB20240279), Jiangsu Funding Program for Excellent Postdoctoral Talent (no.2024ZB209), and the Figures was created with BioRender.com.

## Author contributions

C.L. and Y.T. conducted the majority of the experiments, analyzed data, and wrote the manuscript. R.M. and H.L. wrote the manuscript. T.Y., X.P., R.M., and H.Q. carried out the experiments. All authors read the manuscript and approved the final manuscript.

## Declaration of interests

The authors declare no competing interests.

## STAR★Methods

### Key resources table

**Table:** 

REAGENT or RESOURCE	SOURCE	IDENTIFIER
**Antibodies**		

Anti-Mouse CD25^+^ PE	BioGems	Cat# 07312-60
Anti-Mouse CD3^+^ BG Violet 450	BioGems	Cat# 05121-25
Anti-Mouse CD4^+^ BG Violet 500	BioGems	Cat# 06122-45
Anti-Mouse CD8^+^ FITC	BioGems	Cat# 10112-50
Anti-Mouse/Rat FOXP3 APC	BioGems	Cat# 83422-80
HRP-labelled Goat Anti-Rabbit IgG	Beyotime Biotechnology	Cat# A0208, RRID:AB_2892644
Mouse anti-SREBP1 antibody	Abcam	Cat# ab3259, RRID: AB_1851381
Rabbit anti-Akt antibody	Abcam	Cat# 9272, RRID:AB_329827
Rabbit anti-AMPK antibody	Cell Signaling Technology	Cat# 2532, RRID:AB_330331
Rabbit anti-GADPH antibody	Abcam	Cat# 2118, RRID:AB_561053
Rabbit anti-*p*-Akt (Ser473) antibody	Cell Signaling Technology	Cat# 9271, RRID:AB_329825
Rabbit anti-*p*-AMPK (Thr172) antibody	Cell Signaling Technology	Cat# 2535, RRID:AB_331250
Rabbit anti-PI3K antibody	Abcam	Cat# ab191606, RRID:AB_2891324
Rabbit anti-PPARγ (81B8) antibody	Cell Signaling Technology	Cat# 2443, RRID:AB_823598
Rabbit anti-p-PI3K (Tyr458) antibody	Cell Signaling Technology	Cat# 4228, RRID:AB_659940

**Chemical, peptides, and recombinant proteins**		

A83-01	Biogems	Cat# 9094360
Advanced DMEM/F12	Gibco	Cat# 12634-010
B27	Gibco	Cat# 17504-001
Bovine serum albumin	Sigma	Cat# A9418
Cell recovery solution	Corning	Cat# 354253
Human [leu15]-Gastrin I	Sigma-Aldrich	Cat# G9145
Mogengel Matrix	Mogengel	Cat# 082703
N-acetyl L-cysteine	Sigma-Aldrich	Cat# A9165
Nicotinamide	Sigma-Aldrich	Cat# N0636
Noggin	MCE	Cat# HY-P70558
Prostaglandin E_2_	MCE	Cat# HY-101952
R-spondin-1	MCE	Cat# HY-P7114
SB 202190	MCE	Cat# HY-10295
Wnt3a	MCE	Cat# HY-P70453C

**Deposited data**		

Raw data of Proteome Data	This paper	Mendeley Data, https://doi.org/10.17632/wwgdbwrbxr.1
Raw data of Metabolome Data	This paper	Mendeley Data, https://doi.org/10.17632/r49ggwxbvp.1
Raw data of ITS Data	This paper	Mendeley Data, https://doi.org/10.17632/y75322dtzy.1
Raw data of Western Blot Data	This paper	Mendeley Data, https://doi.org/10.17632/3fzfd2npw8.1

**Critical commercial assays**		

All-in-one RT SuperMix Perfect for qPCR	Vazyme Biotech	Cat# R333
Cell/Tissue Total RNA Isolation Kit	Vazyme Biotech	Cat# RC112
Spin Column Animal Total RNA Purification Kit	Sangon Biotech	Cat# B518651
SYBR qPCR Master Mix	Vazyme Biotech	Cat# Q712

**Experimental models: Organisms/strains**		

C57BL/6J (male)	GemPharmatch	Strain NO.N000013
C57BL/6J ApoE^−/−^(male)	GemPharmatch	Strain NO.T001458

**Software and algorithms**		

DAVID	DAVID Team	RRID:SCR_001881
FLowJo v10.8	Tree Star	RRID: SCR_008520
GraphPad Prism 7.0	GraphPad software	RRID: SCR_002798
ImageJ	NIH	RRID: SCR_003070
Metaboanalyst 5.0	NIH-Canada Research	RRID: SCR_015539
Metascape	NIH-metascape.team	RRID:SCR_016620
RStudio	RStudio, Inc	RRID:SCR_000432

### Experimental model and study participant details

C57BL/6J and C57BL/6J ApoE^−/−^ Male mice, aged 6 weeks, were procured from Gem Pharmatech Co., Ltd. (Nanjing, China). Mice were housed in a specific pathogen-free facility with a 12-h light-dark cycle, humidity ranging from 40% to 60%, and room temperature maintained between 24°C and 26°C. They had *ad libitum* access to diet and water. All experimental procedures and protocols using mice were approved by the Animal Ethics Committee of Jiangnan University (JN. No20210630c1401201[267]) and were carried out in accordance with the approved institutional guidelines and regulations.

Fecal samples were collected from 3 male volunteers, aged 27–29 years, with serum total cholesterol >5.7 mmol/L and triglycerides >1.7 mmol/L, who had not taken antibiotics for six months, under approval from the Medical Ethics Committee of Jiangnan University (JNU202312IRB11). All research was conducted in accordance with relevant guidelines and regulations, and written informed consent was obtained from all volunteers. Table S1 provides clinical details.

### Method details

#### Establishment of the obese mouse model and Akk intervention

The Akk strain (CICC 24917) was cultured anaerobically in a medium containing 3.85% brain heart infusion, 0.5% porcine mucin, and 0.05% L-cysteine. The culture underwent centrifugation, washing, and resuspension in anaerobic PBS to achieve a final concentration of 1 × 10^9^ cfu/mL.

The experimental procedure is depicted in Figure 1A. Twenty-four male C57BL/6J ApoE^−/−^ mice, aged 6 weeks, were procured from Gem Pharmatech Co., Ltd. (Nanjing, China). After 7 days of adaptation period, all mice, except for the control group, were fed a high-fat, high-cholesterol diet (HFHCD) from Week 1 to Week 20. From Week 11 to Week 20, Akk group (**Akk**) was orally administered 200 μL of bacterial suspension every day, while the control (**Con**) and model (**Mod**) groups were given saline. The control group was maintained on a standard diet, whereas the experimental groups were given an HFHCD diet composed of 21% fat (42% of calories), 21% protein (19% of calories), 43% carbohydrates (38% of calories), and 0.5% cholesterol.

#### Analysis of Pathological and inflammatory markers

Blood samples were collected followed by the prompt excision and imaging of the intact livers. Tissue homogenates were prepared by homogenizing in PBS and then centrifuging at 3000 × g for 10 min at 4°C. The concentrations of proteins, IL-6, IL-1β, IL-10, and IL-17 in the serum and tissue homogenate supernatants were quantified using ELISA kits, following the manufacturer’s protocols (Nanjing SenBeiJia Biological Technology Co., Ltd.).

#### Detection of fungi

DNA was extracted using a DNA purification kit. The fungal-specific primers used were ITS5-1737F (GGAAGTAAAAGTCGTAA CAAGG) and ITS2-2043R (GCTGCGTTCTTCATCGATGC), targeting the ITS1-5F region. The PCR conditions included an initial denaturation at 95°C for 2 min, followed by 25 cycles of 95°C for 30 s, 55°C for 30 s, and 72°C for 30 s, with a final extension step at 72°C for 5 min. The fungal ITS rRNA sequences were then analyzed using the UNITE database for sequence alignment and identification.

Sequencing and data processing involved verification of amplification products through 2% agarose gel electrophoresis, purification, and quantification. Sequencing of qualified libraries was performed on the Novaseq 6000 PE250 (Illumina Inc., San Diego, USA) platform. Species annotation and visualization utilized representative sequences and feature tables, with RDP classifier and the UNITE database for fungal ITS rRNA.

#### Analysis of flow cytometry

Antibodies for CD3 (Cat# 05121), CD4 (Cat# 06122), CD8 (Cat# 10112), CD25 (Cat# 07312), FOXP3 (Cat# 83422), and the fixation/permeabilization kit (Cat# 92550:92160, 1:3, v/v) were obtained from BioGems (USA). Intestinal lymph nodes from the mice were harvested and homogenized in 3 mL of ice-cold PBS. The homogenate was passed through a 70 μm strainer to create a single-cell suspension, which was then centrifuged at 600 × g for 8 min at 4°C. The supernatant was discarded, and the cell pellet was resuspended in 5 mL of precooled red blood cell lysis buffer, followed by gentle mixing and an 8-min incubation at room temperature. The reaction was stopped by adding 10 mL of PBS, and suspension was centrifuged again at 600 × g for 8 min at 4°C. This lysis and wash step was repeated once.

The resulting cell pellet was resuspended in PBS to achieve a concentration of 2 × 10^7^ cells/mL. A 500 μL aliquot of this suspension was incubated with the following antibodies: CD3^+^ (Violet 450), CD4^+^ (Violet 500), CD8^+^ (FITC), and CD25^+^ (PE). The cells were incubated in the dark at room temperature for 25 min, followed by centrifugation at 500 × g for 8 min. The supernatant was removed, and 1 mL of freshly prepared fixation/permeabilization solution was added. The mixture was incubated in the dark at room temperature for 30 min. Cells were centrifuged at 800 × g for 8 min, the supernatant discarded, and 1 mL of permeabilization buffer was added. After dispersing the cells, they were incubated in the dark at room temperature for 15 min, followed by centrifugation at 800 × g for 8 min. The cell pellet was resuspended in 100 μL of permeabilization buffer (1X) with the Foxp3^+^ antibody (APC) and incubated in the dark at room temperature for 15 min. The cells were then washed with 1 mL of permeabilization buffer (1X), centrifuged at 800 × g for 8 min, and resuspended in 500 μL of PBS for analysis.

Flow cytometry data were analyzed using FlowJo v10.8 (Tree Star, USA). Preprocessing steps included quality control checks to assess the integrity of the data, normalization of cell counts using the Downsample method, and noise reduction via quantile gating. For data characterization and population identification, clustering was performed using the FlowSOM.^40^ Algorithm, and nonlinear dimensionality reduction was applied using the PHATE method.^41^ These advanced computational techniques are widely adopted for analyzing complex flow cytometry data and allow for accurate identification of immune subsets, including Tregs, based on multi-parameter marker expression.

#### Analysis of metabolomics

Sample extraction was performed as described in a previous study.^42^ Briefly, 100 μL or mg of sample was transferred into a tube, and 200 μL of methanol containing 1 μg/mL of an internal standard was added. The mixture was vortexed for 15 min to ensure thorough mixing. The extract was then centrifuged at 20,000 × g for 10 min at 4°C. The supernatant was collected and dried under vacuum. The dried extracts were resuspended in 200 μL of ice-cold methanol and sonicated in an ice bath for 10 min to ensure proper dispersion before proceeding to LC-MS analysis, which consisted of a UPLC (AB SCIEX ExionLC) and a TOFMS (AB SClEX, Triple TOF 5600^+^).

Peak detection conditions were 20 average peak width scans and 10,000 minimum peak height scans. Deconvolution was performed using a 0.5 Sigma window value and an electron ionization spectrum cutoff of 5,000 amplitudes. The retention time was controlled within 0.5 min, the m/z identification tolerance was 0.5 Da, the electron ionization similarity cutoff was 70%, and the identification score cutoff was 70%. The retention time tolerance was 0.075 min, and the retention time factor was 0.5 when setting the calibration parameters. Data processing steps included data normalization, metabolite content statistics, unsupervised dimensionality reduction analysis, and analysis and screening of characteristic metabolites. In addition, pathway analysis (enrichment analysis, metabolic pathways) of characteristic metabolites was completed on the MetaboAnalyst 5.0 Website.

#### Analysis of proteomics

For the proteomics analysis, a Data-Independent Acquisition (DIA) method was used.^43^ Protein extraction and digestion were performed by first pre-freezing mouse liver samples in liquid nitrogen and then grinding them into powder. The powder was mixed with lysis buffer (7 mol/L urea, 2 mol/L thiourea, 1 g/L CHAPS, protease inhibitors) at a 1:10 ratio (w/v), homogenized, and incubated on ice for 30 min. The homogenate was centrifuged at 13,000 × g for 20 min at 4°C, and the supernatant was stored at −80°C. Protein concentration was measured using the Bradford method. For digestion, 10 μg of protein from each sample was pooled (totaling 250–300 μg) for database creation, and 20 μg was set aside for DIA. Proteins were reduced with 0.1 mol/L DTT (25 mmol/L final concentration) at 37°C for 1 h, alkylated with 0.5 mol/L IAA (50 mmol/L final concentration) in the dark for 30 min, and centrifuged at 13,000 × g for 10 min. After washing three times with 50 mmol/L NH_4_HCO_3_, trypsin was added at a 1:50 ratio and incubated overnight at 37°C.

Then, peptide desalting and separation were performed. Digested peptides were desalted using HLB solid-phase extraction columns for database establishment, and custom-made C_18_ tip columns for the DIA experiment. Desalted peptides were dissolved in buffer A (98% deionized water, 2% acetonitrile, pH 10) and separated using an offline XBridge Peptide BEH C_18_ HPLC column with buffer B (98% acetonitrile, 2% deionized water, pH 10). Peptides were eluted at 1 mL/min over 51 min, collecting fractions every minute into 40 tubes. Fractions were dried under vacuum and stored at −20°C.

LC-MS/MS analysis and data processing were subsequently conducted. Peptides were redissolved in 11 μL of 1 mL/L formic acid solution with 1 μL of iRT peptides per tube. A 1 μL aliquot from each tube was used for quality control. Peptides were separated using a low-pH reverse-phase C18 capillary column and eluted with a gradient of 5%–38% over 90 min. They were analyzed using an Orbitrap Exploris 240 mass spectrometer in high-sensitivity mode. Full scans were acquired at 60,000 resolutions, and MS/MS scans at 15,000 resolutions, covering a mass range of 350–1500 m/z for MS and 110–1500 m/z for MS/MS, with an HCD collision energy of 38%. Data were processed using Proteome Discoverer (version 2.3) and Spectronaut Pulsar (version 15.0), with the uniprot-mouse_55341_20211214 databases. Parameters included trypsin digestion with up to two missed cleavages, 10 ppm mass tolerance for precursor ions, and 0.02 Da for-fragment ions. Fixed modification was carbamidomethylation; variable modifications were oxidation and N-terminal acetylation. Peptide and protein false discovery rates were set to <1.0%, and DIA data were matched to the spectral library with a q-value <1.0%.

#### Co-culture of fecal microbiota from patients with NAFLD, colonic organoids, and Akk *in vitro*

As shown in Figure 7A. Fecal samples were collected from volunteers. Crypts were isolated from colons of 4-6-week-old C57BL/6 mice.^44^^,^^45^ Colons were cut, washed with cold DPBS, and treated with 2 mM EDTA/DPBS at 4°C for 30 min. The tissue was disrupted using a 1 mL pipette rinsed with cold 0.1% BSA/DPBS, then dispersed in 0.1% BSA/DPBS. The final cleaning solutions were filtered through a 70 μm filter, centrifuged at 200 × g for 3 min at 4°C, and crypts were resuspended in matrix glue. A total of 200 crypts in 50 μL matrix glue were plated in a 24-well plate (50 μL/well) and incubated at 37°C for 30 min to solidify. After 3 days of subculturing, the differentiation medium was replaced, and fecal microbiota solution (10%) was added at a 1:1 volume ratio. The experimental groups included: **con** (intestinal organoids without fecal microbiota or Akk), **vehicle** (intestinal organoids with fecal bacteria), and **Akk** (intestinal organoids with both fecal microbiota or Akk at 10^6^ cfu/mL). The differentiation cycle lasted 4–6 days, with daily replacement of the culture medium, fecal bacteria, and fecal microbiota or Akk. The organoid was observed using DAPI staining to assess cell viability and nuclear integrity.

To selectively remove gut fungi, fluconazole (30 μg/mL) was added to the medium in the relevant groups, ensuring that the fungi were specifically depleted without affecting the bacterial population. The experimental groups included: **con’** (intestinal organoids without fecal microbiota or Akk), **vehicle’** (intestinal organoids with fecal bacteria, and fluconazole), and **Akk’** (intestinal organoids with both fecal microbiota or Akk at 10^6^ cfu/mL, and fluconazole).

#### Macrophage lipid metabolism assay

As shown in Figure 8A, following the protocol from a prior study.^46^ RAW 264.7 mouse macrophage cells were cultured in DMEM supplemented with fetal bovine serum, penicillin, and streptomycin (37°C, 5% CO_2_). The cell line was authenticated by PCR and tested negative for mycoplasma contamination. The medium was refreshed every 1–2 days. Once the cells reached 80%–90% confluence, they were passaged at a ratio of 1:3. The cells were then seeded into a 24-well plate at a density of 1 × 10^5^ cells/well. After 24 h of incubation, the cells were treated with two metabolites, glycylleucine and α-Ketoisovaleric acid, each at a final concentration of 20 μg/mL, and ox-LDL at a final concentration of 100 μg/mL, for an additional 24 h. In the model group (**Mod**), PBS and ox-LDL were added to maintain the same final volume, while the control group (**Con**) received only PBS to ensure consistent final volumes.

For Oil Red O staining, the cells were washed three times with PBS and fixed with 10% formalin for 30 min. They were then stained with Oil Red O solution (0.5% w/v in isopropanol) for 30 min. After staining, the cells were washed three times with PBS to remove excess stain and imaged under an optical microscope.

#### α-Ketoisovaleric acid compensation experiment in mice

As shown in Figure 9A and 24 male C57BL/6J mice, aged 6 weeks, were raised for 7 days of adaptation period. All mice, except for the control group, were fed an HFHCD from Week 1 to Week 8. At the same time, the control group (**Con**) and the model group (**Mod**) were given saline by gavage, while the α-Ketoisovaleric acid group (50 mg/kg) were fed sample.

#### Western blotting

Mouse tissues were homogenized in RIPA lysis buffer containing phosphatase and protease inhibitors, and protein concentration was measured. Equal amounts of protein were separated by 10% SDS-PAGE and transferred onto PVDF membranes. The membranes were blocked with 5% non-fat milk in TBST buffer at room temperature for 2 h. They were then incubated overnight at 4°C with primary antibodies against PI3K (1:1000), Akt (1:1000), GAPDH (1:2000), PPARγ (1:1000), SREBP-1c (1:1000), p-PI3K (1:1000), *p*-Akt (1:1000), AMPK (1:1000), *p*-AMPK (1:1000). After washing, the membranes were incubated with secondary antibodies at room temperature for 2 h and washed five times with TBST. Protein bands were detected using enhanced chemiluminescence reagents and quantified using ImageJ software. Antibodies were obtained from Abcam and Cell Signaling Technology.

#### Real-time quantitative PCR

Total RNA was extracted from samples and quantified using a spectrophotometer. Samples with an absorbance ratio of 260/280 nm between 1.8 and 2.0 were diluted to the same concentration. RNA was reverse transcribed into cDNA using a cDNA synthesis kit. RT-qPCR was performed using SYBR qPCR Master Mix and a real-time PCR system. Kits for cDNA synthesis (R333) and SYBR qPCR Master Mix (Q712) were obtained from Vazyme (Nanjing, China). Relative gene expression was calculated using the 2^−ΔΔCt^ method. Primer sequences are provided in the Table S2.

### Quantification and statistical analysis

All experiments were conducted at least three times, and data are presented as mean ± standard deviation (SD). Statistical analysis was performed using GraphPad Prism 10.0 (GraphPad Prism Software Inc., San Diego, California). For comparisons among multiple groups, one-way ANOVA followed by Dunnett’s post-hoc test was employed. Linear discriminant analysis (LDA) was used for dimensionality reduction and evaluation of differences. Correlation analysis (with coefficients ranging from −1 to 1) was conducted using Spearman’s method and visualized with R software (Vienna, Austria). Structural equation modeling was carried out using AMOS software, specifying a hypothesized model with latent and observed variables.

## Supplemental information

Document S1. Figures S1–S3

Table S1. Clinical details

Table S2. Primer sequence of RT-qPCR
