# Supplementary material for: Akkermansia muciniphila ameliorates fatty liver through microbiota-derived α-ketoisovaleric acid metabolism and hepatic PI3K/Akt signaling
Source: iScience. 2025 Apr 16;28(5):112458. doi: 10.1016/j.isci.2025.112458 (PMC12059670; doi:10.1016/j.isci.2025.112458)

iScience, Volume *28*

Supplemental information

Akkermansia muciniphila ameliorates fatty liver through microbiota-derived α-ketoisovaleric acid metabolism and hepatic PI3K/Akt signaling

Chang Liu, Rongrong Ma, Han Li, Xiaohua Pan, He Qian, Tianyi Yang, and Yaoqi Tian

Supplemental information

Fig.S1 Network interaction diagram of differentially expressed proteins via STRING enrichment website

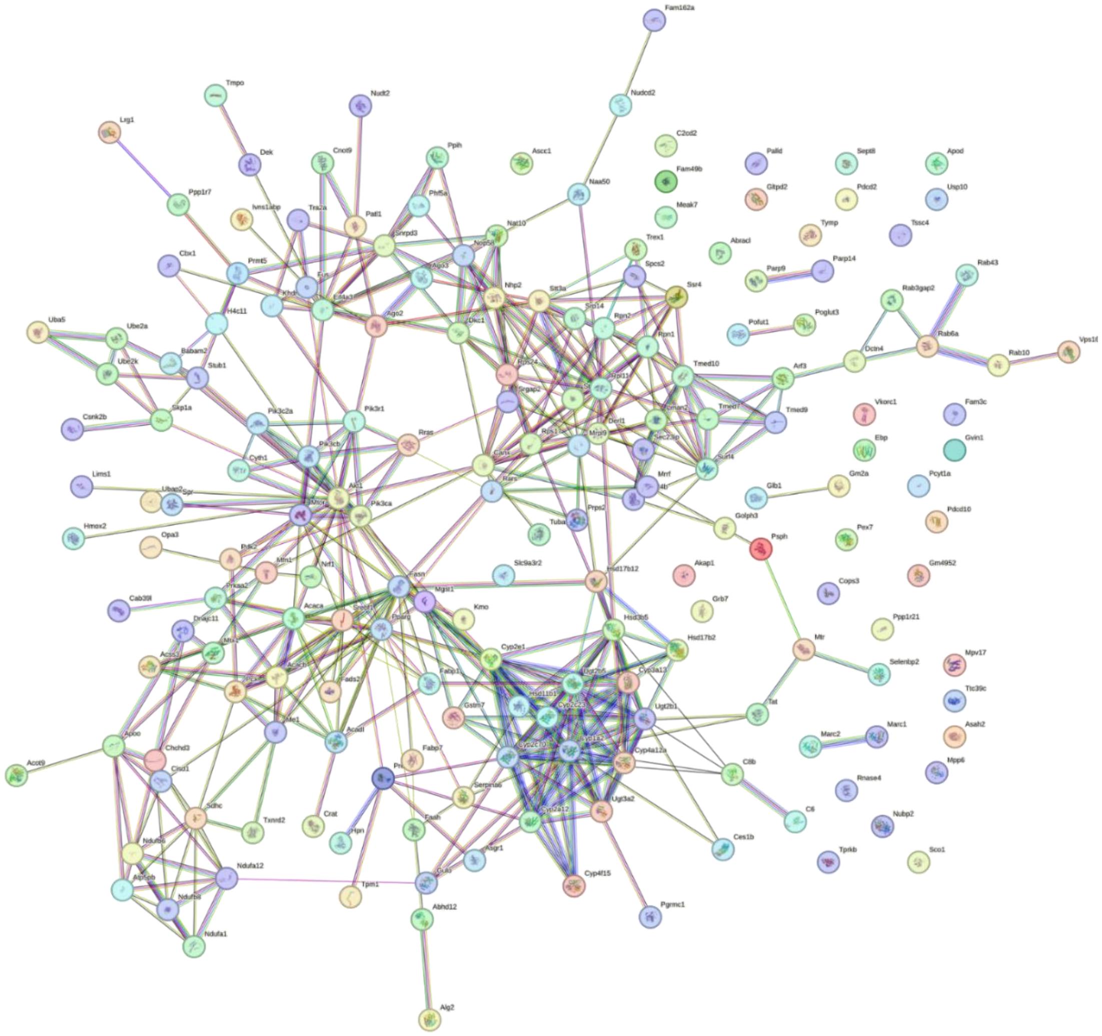

Fig.S2 Regression Analysis of Key Metabolites Regulated by Akk with Gut Fungi

(A-D) Regression analysis of 3-Methyl-2-oxovaleric acid, α-Ketoisovaleric acid, glycylleucine, and caffeine with *Archaeorhizomyces*.

(E、G) Regression analysis of α-Ketoisovaleric acid and caffeine with *Saccharomycopsis*.

(F、H) Regression analysis of 3-Methyl-2-oxovaleric acid and glycylleucine with *Saccharomycopsis*

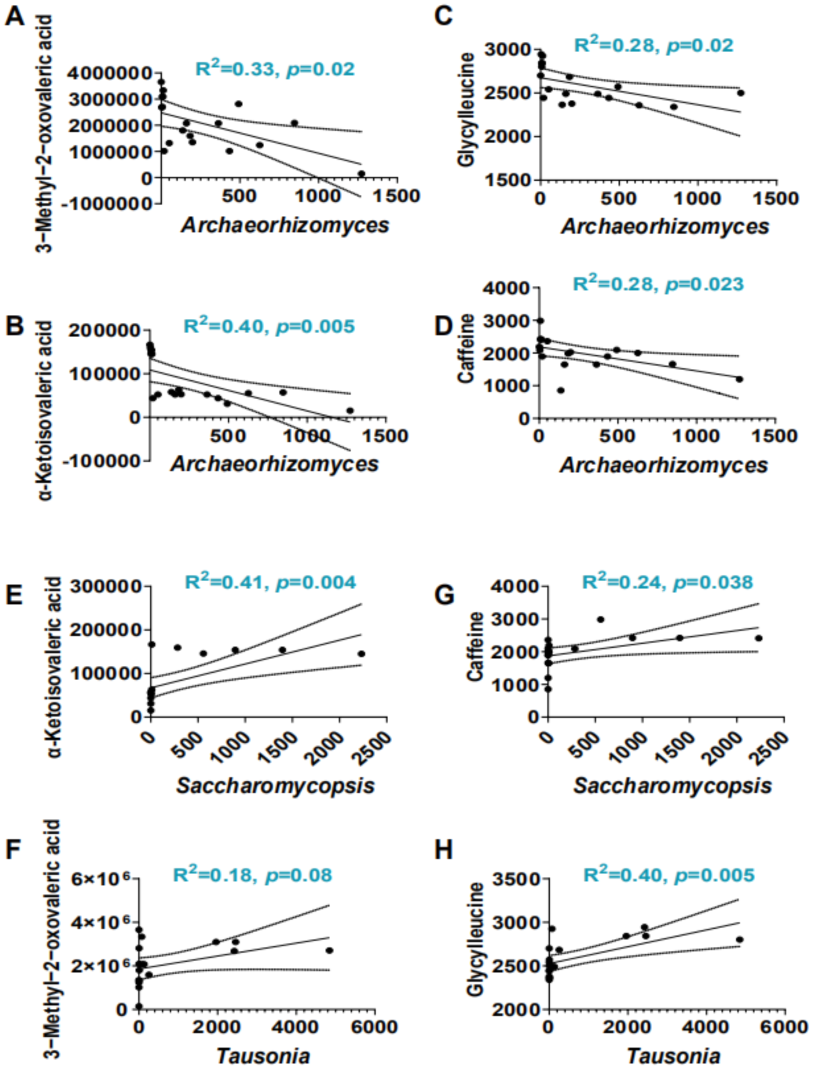

Fig.S3 Effects of Akk and α-Ketoisovaleric acid on CD36 gene in colon and liver

(A-B) mRNA levels of genes related to CD36 in mouse colon and liver with the intervention of Akk.

(C-D) mRNA levels of genes related to CD36 in mouse colon and liver with the intervention of α-Ketoisovaleric acid.

Data are expressed as mean ± SD (n=6-8), *p < 0.05, **p < 0.01, ***p < 0.001, compared with the Mod group, and p-value was calculated using Dunnet's post hoc test in one-way ANOVA.

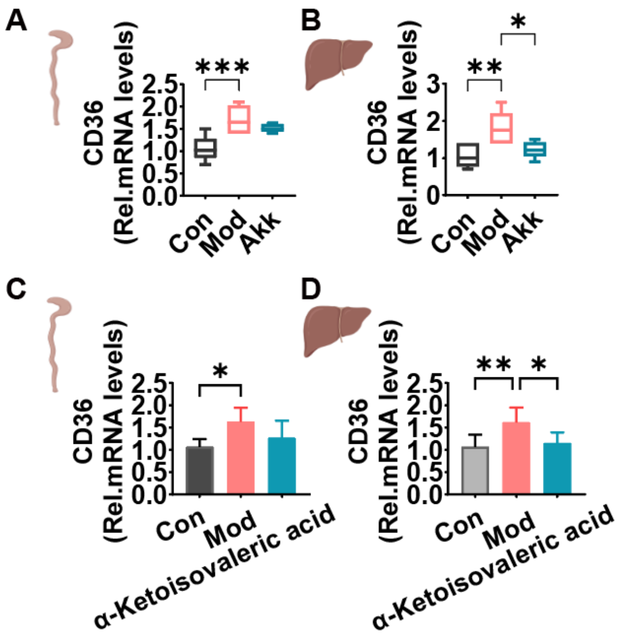

Supplement: Document S1. Figures S1–S3 [file mmc1.pdf]
